# Silencing of an Ubiquitin Ligase Increases Grain Width and Weight in *indica* Rice

**DOI:** 10.3389/fgene.2020.600378

**Published:** 2021-01-12

**Authors:** Ankit Verma, Geeta Prakash, Rajeev Ranjan, Akhilesh K. Tyagi, Pinky Agarwal

**Affiliations:** ^1^National Institute of Plant Genome Research, New Delhi, India; ^2^Department of Botany, Gargi College, University of Delhi, New Delhi, India; ^3^Department of Plant Molecular Biology, University of Delhi, New Delhi, India

**Keywords:** E3 ubiquitin ligase, grain width, GW2, *Oryza sativa*, seed development

## Abstract

Many quantitative trait loci (QTLs) have been identified by molecular genetic studies which control grain size by regulating grain width, length, and/or thickness. *Grain width 2* (*GW2*) is one such QTL that codes for a RING-type E3 ubiquitin ligase and increases grain size by regulating grain width through ubiquitin-mediated degradation of unknown substrates. A natural variation (single-nucleotide polymorphism at the 346^th^ position) in the functional domain-coding region of *OsGW2* in *japonica* rice genotypes has been shown to cause an increase in grain width/weight in rice. However, this variation is absent in *indica* rice genotypes. In this study, we report that reduced expression of *OsGW2* can alter grain size, even though natural sequence variation is not responsible for increased grain size in *indica* rice genotypes. *OsGW2* shows high expression in seed development stages and the protein localizes to the nucleus and cytoplasm. Downregulation of *OsGW2* by RNAi technology results in wider and heavier grains. Microscopic observation of grain morphology suggests that OsGW2 determines grain size by influencing both cell expansion and cell proliferation in spikelet hull. Using transcriptome analysis, upregulated genes related to grain size regulation have been identified among 1,426 differentially expressed genes in an *OsGW2*_RNAi transgenic line. These results reveal that *OsGW2* is a negative regulator of grain size in *indica* rice and affects both cell number and cell size in spikelet hull.

## Introduction

Globally, rice (*Oryza sativa* L.) is a staple cereal food crop since it is the primary source of caloric intake and is consumed by more than half of the world's population (Sasaki, 2008; Zuo and Li, 2014; Azizi et al., 2019). In upcoming decades, the overall demand of rice will increase, particularly in Asia and Africa (Kubo and Purevdorj, 2004). Hence, there is an urgent need to increase rice grain yield. Rice grain size/weight is the key agronomic trait for the improvement of yield. Rice grain yield is a complex trait, which is determined by four typical quantitative component traits, i.e., number of panicles per plant, number of filled grains per panicle, grain size, and grain weight (Xing and Zhang, 2010; Zuo and Li, 2014). Grain size (grain length and width)/weight is the most vital yield-contributing complex quantitative trait in rice (Fan et al., 2006; Mao et al., 2010; Ying et al., 2012). Therefore, the best approach for enhancing rice yield is to find/develop new high yielding varieties with increased grain size/weight and superior grain nutrient quality (Rosegrant and Cline, 2003). Grain weight and grain size are positively associated with each other. Grain size is defined in terms of its length, width, and thickness. Rice grain size is mainly controlled by genes that determine cell expansion and/or proliferation in spikelet hull and contribute in endosperm development. All these yield component traits are controlled by naturally occurring quantitative trait loci, QTLs (Zuo and Li, 2014). Several QTLs and a number of genes regulating grain size have been identified and functionally characterized by different approaches such as genomics, proteomics, metabolomics, genome editing, and genome-wide association to find out molecular components and genetic regulatory mechanisms controlling grain size trait in rice (Li and Li, 2014; Li et al., 2018; Azizi et al., 2019). Hitherto, much needs to be explored regarding the molecular mechanisms as well as the underlying genes controlling rice grain size/weight to understand the mechanism of grain development, which will provide a way to improve yield and quality.

Ubiquitination has been shown to play a significant role in determining seed size in crop plants (Song et al., 2007; Li et al., 2008; Choi et al., 2018). It is an enzymatic post-translational modification in which ubiquitin protein (76 residues long) covalently attaches with a target protein (Li and Li, 2014), with the consecutive participation of three unique enzymes, E1, E2, and E3 (Moon et al., 2004). E1 is a ubiquitin-activating enzyme. E2 conjugates with activated ubiquitin. Ubiquitin ligase E3 determines substrate specificity (Smalle and Vierstra, 2004). This process regulates the stability, activity, and localization of modified target proteins. Mono-ubiquitination of a target protein affects its function and localization, whereas conjugation of multi-ubiquitin can degrade modified proteins through the ubiquitin−26S proteasome complex (Vierstra, 2009).

Grain yield and quality are controlled by several QTLs and genes. A number of grain size related QTLs (*GW2, GW5, GS3, GS5, qGL3, TGW6, GW8*, etc.) and genes have been functionally characterized in the last few decades. They regulate either cell division or cell expansion, or both, during the process of seed development, by being involved in signaling pathways facilitated by ubiquitin-mediated proteasomal degradation, transcription factors, guanine nucleotide-binding proteins (G-proteins), protein kinases and phytohormones (Zuo and Li, 2014; Wang et al., 2019; Zhao et al., 2019). *GW2* is one such QTL that regulates grain weight/yield by regulating grain width in *japonica* rice genotypes. It codes for a RING-type protein having E3 ubiquitin ligase activity that binds with its substrates and targets them for degradation through the ubiquitin−26S proteasomal complex. A natural variation in long grain WY3 cultivar results in truncation of the functional domain of GW2. This results in a wider spikelet hull and an increase in the number of cells in the outer parenchyma cell layer, without affecting cell numbers in the endosperm. Hence, it has been identified that *GW2* negatively regulates grain size and weight in *japonica* rice cultivars (Song et al., 2007). Chitinase 14 (CHT14) and phosphoglycerate kinase (PGK) are involved in carbohydrate metabolism and show strong interaction with GW2, but are not involved in ubiquitin-mediated degradation (Lee et al., 2018). GW2 also targets expansin-like 1 (EXPLA1), which is a cell wall-releasing protein, for degradation through the proteasome pathway and hence participates in determining seed size and weight (Choi et al., 2018). WIDE AND THICK GRAIN 1 (WTG1) also controls rice grain size through the ubiquitin–proteasome pathway. WTG1 is a deubiquitinating enzyme, an otubain-like protease. WTG1/OsOTUB1 regulates seed size/shape by affecting only cell expansion in the hull, suggesting a different pathway than GW2 (Jiao et al., 2010; Huang et al., 2017; Li and Li, 2019). DA2 is the homolog of GW2 in *Arabidopsis thaliana* that also controls seed and organ size. Rice GW2 homologs have been identified in maize and wheat, where they regulate kernel size (Li et al., 2010; Sestili et al., 2019).

Although the specific natural variation in *GW2*, causing its inactivation, results in higher grain width/weight in *japonica* rice genotypes, such a sequence variation is absent in *indica* genotypes (Dixit et al., 2013). The aim of the current study was to examine if, despite the absence of this variation, downregulation of *GW2* in *indica* rice would alter grain size. Downregulation in transgenic rice plants, by RNAi, revealed that the grain width/weight in *indica* rice genotypes increases due to higher cell division as well as expansion in spikelet hull which in turn is controlled by differential gene expression of many seed development-related genes.

## Materials and Methods

### Plant Material and Growth Conditions

Rice *indica* and *japonica* genotypes (PB1, IR64, Sonasal, LGR, and Nipponbare) were grown in experimental fields of NIPGR (New Delhi, India) during the months of June to October (*T*_max_ 35–40°C; *T*_min_ 25–28°C). Rice transgenic plants along with the wild-type lines (WT) were grown in a mix of soil and compost (3:2), which was supplemented with NPK, in a greenhouse with a 12-h/12-h light/dark cycle, at 28/23°C temperature, 65% relative humidity, and 250 μmol m^−2^ s^−1^ photosynthetic active radiation (PAR) light. The complete development of rice seed occurs in five different stages based on morphological changes occurring in the embryo and endosperm categorized as S1 (0–2 DAP, days after pollination), S2 (3–4 DAP), S3 (5–10 DAP), S4 (11–20 DAP), and S5 (21–29 DAP) (Agarwal et al., 2007, 2011). In accordance, rice panicles were tagged and harvested as mentioned previously (Sharma et al., 2012). Flag leaf and seed tissue from each variety, at each stage, were harvested and frozen in liquid nitrogen and stored at −80°C until further use.

### RNA Isolation, cDNA Preparation, and Quantitative RT-PCR

Isolation of RNA from the leaves and seeds (S1–S5 stages) of different rice genotypes (PB1, IR64, Sonasal, LGR, and Nipponbare) as well as transgenic plants was carried out using TRIzol® reagent (Invitrogen^TM^ Life Technologies, USA) as detailed previously (Singh et al., 2003; Mathew et al., 2016, 2020; Das et al., 2019). The quantification of RNA samples was done by NANODROP 2000c Spectrophotometer (Thermo Scientific, USA). For the amplification of the *OsGW2* gene (LOC_Os02g14720), full-length cDNAs of five rice genotypes, mentioned above, were used. For full-length cDNA synthesis, 2 μg of purified RNA and Superscript III® first-strand cDNA synthesis kit (Invitrogen^TM^ Life Technologies, USA) was used. Reverse transcription of purified RNA was performed with High Capacity cDNA Reverse Transcription Kit (Applied Biosystems, USA) with random primers. Gene-specific primers were designed by using Primer Express Version 3.0 (Applied Biosystems, USA). For expression analysis of *OsGW2*, qRT-PCR was performed in three biological replicates using gene-specific primers (Supplementary Table 1), with Fast SYBR® Green Master Mix (Applied Biosystems, USA). Real-time PCR was done in 7500 Fast Real-Time System (Applied Biosystems, USA) using KAPA SYBR® FAST qPCR kit and 7500 software v2.0.1 for data analysis. *OsACTIN1* gene was the endogenous control for leaf and seed tissue throughout the study. Relative gene expression level was calculated by the comparative Ct (2^−ΔΔCt^) method.

### Sequence Analysis of *OsGW2* in Different Rice Genotypes

The *OsGW2* gene coding region was amplified from different rice genotypes (IR64, Sonasal, LGR, and PB1) using full-length cDNA for each (Supplementary Table 1) and cloned in pJET^1.2^ vector (Thermo Scientific, USA). Cloning was confirmed by restriction digestion by *Bgl*II and confirmed clones were sequenced on ABI 3730xl DNA Analyzer (Applied Biosystems, USA) in three replicates. The obtained sequences were analyzed by Clustal Omega tool (Sievers et al., 2011) for aligning the insert sequence with template sequence extracted from the Rice Genome Annotation Project (RGAP, http://rice.plantbiology.msu.edu/). Any difference in either nucleotide or subsequent protein sequence was noted.

### Preparations of RNAi Construct and Rice Plant Transformation

To generate the RNAi construct, a 510-bp unique region of *OsGW2* was delineated by Rational siRNA Design program (Reynolds et al., 2004). This unique region of *OsGW2* was amplified along with CACC sequence added to the 5′ end of the forward primer (Supplementary Table 1), from the *OsGW2*_pJET clone. The amplified fragment was purified using GeneJET gel extraction kit (Thermo Scientific, USA), followed by cloning into Gateway® entry vector pENTR^TM^/D-TOPO® (Invitrogen^TM^, USA) containing recombination sites (*attL1* and *attL2*) using pENTR^TM^/Directional TOPO® cloning kit as per the manufacturer's protocol. Cloning was confirmed by restriction digestion with *Asc*I and *Not*I restriction enzymes and sequencing of two positive clones. Plants with reduced expression or knockdown of *OsGW2* were made using RNAi vector pANDA (Miki and Shimamoto, 2004), in which transgene expression was under the control of the maize *UBIQUITIN* promoter. The insert was transferred to pANDA having kanamycin and hygromycin selection marker genes using Gateway^TM^ LR Clonase^TM^ II Enzyme Mix (Invitrogen^TM^, USA) through Gateway® cloning technology. Target sequence was incorporated in opposite orientation in two *attR* sites located at both sides of the *GUS* linker sequence, confirmed by restriction digestion by using *Kpn*I and *Sac*I enzymes. The confirmed clone was introduced into the *Agrobacterium tumefaciens* strain *EHA105*. Transgenic plants were generated by transformation of the RNAi construct into rice calli (PB1 genotype IET-10364) through the *Agrobacterium*-mediated transformation method (Toki et al., 2006; Das et al., 2019; Mathew et al., 2020).

### Phenotypic Characterization of Rice Transgenic Plants

Vegetative phenotypic characters (plant height, flag leaf length and width, number of tillers per plant, number of panicles per plant, and number of grains per panicle) were measured manually and compared with the respective wild-type plant in T_0_, T_1_, and T_2_ generations. Six biological replicates were taken to quantitate each vegetative character for each line. Grain parameters (length and width) were measured after the grains were harvested and dried, by using WinSEEDLE^TM^ (Regent Instruments Inc., Canada). Photographs of grain length and width were taken using a Nikon D5200 48 MP DSLR camera.

### Scanning Electron Microscopy

For scanning electron microscopy analysis, the mature grains of homozygous T_2_ transgenic line *OsGW2*_RNAi_7AP9 and wild-type plants were harvested. To study cell size and number in the outer epidermis, a scanning electron microscope (SEM, Zeiss, Germany) was used to observe the central parts of the lemma surface of mature grains, under different magnifications (500×, 1,000×, and 2,500×). For analyzing starch granules, mature rice grains were dehusked manually. These were sectioned longitudinally with a scalpel and the central parts of the endosperm were photographed by SEM. To analyze cell length and cell area of the outer epidermal cells, ImageJ software was used (Schneider et al., 2012).

### Subcellular Localization of OsGW2

The full-length coding sequence of *OsGW2* from PB1, lacking the stop codon, was amplified (Supplementary Table 1) and cloned downstream of the YFP coding region in the pSITE-3CA-YFP vector (Chakrabarty et al., 2007) under the control of CaMV *35S* promoter to generate the recombinant plasmid YFP-OsGW2. To study the subcellular localization of OsGW2, YFP-OsGW2 recombinant plasmid and control YFP vector were bombarded separately into onion peel cells by particle bombardment using Biolistic®-PDS-1000/He^TM^ system (Bio-Rad, USA). After incubation for 16 h in the dark at 28°C, YFP fluorescence was observed with a confocal laser microscope (TCS SP2, Leica, Germany).

### Transcriptome Analysis of *OsGW2*_RNAi Plants

To study the functional relevance of *OsGW2*, RNA-seq analysis of T_3_ grains, from S4 stage, of *OsGW2*_RNAi_7AP9 transgenic plants, along with the wild-type lines, was performed in two biological replicates. RNA isolation and quantification was done as mentioned above and RNA integrity was checked on Bioanalyzer (2100 Agilent Technologies, USA). RNA having RIN ≥8.0 were used for library preparation. Total RNA (~5 μg) for each sample was used for cDNA library preparation and sequencing. Transcriptome sequencing was performed on Illumina Hiseq^TM^ 2000 platform with two independent biological replicates, each for WT and *OsGW2*_RNAi_7AP9. After obtaining sequencing data, different tools were used for performing preprocessing of data, alignment with reference, expression estimation, and comparison analysis, as detailed previously (Mathew et al., 2020). The preprocessing tools used were Adapter Removal v2 (version 2.2.0) and bowtie2 (version 2.2.9). Processed reads were aligned in STAR (version 2.5.3a) with the MSU rice genome database (ftp://ftp.plantbiology.msu.edu/pub/data/Eukaryotic_Projects/o_sativa/annotation_dbs/pseudomolecules/version_7.0/all.dir). Cufflinks was used for estimating gene expression (version 2.2.1) and output was in terms of fragments per kilobase million reads (FPKM). To identify differentially expressed genes (DEGs), Cuffdiff utility provided in Cufflinks package was used. The log_2_ fold change cutoff was ≥1.5 (upregulated genes) and ≤−1.5 (downregulated genes) with a *P-*value cutoff of ≤ 0.05.

Functional annotation of DEGs in metabolic pathways/processes was studied by using databases including the KEGG pathway analysis and MSU-RGAP 7 (Ouyang et al., 2006). Characterized differentially expressed genes were separated and analyzed using RICENCODE, and seed-related genes were extracted by comparing DEGs with the data available from previous studies in the lab and online sources, as done previously (Mathew et al., 2020). Heat maps were constructed using MeV Version 4.9.0 (Saeed et al., 2003). Gene ontology enrichment for various DEGs was performed using the AgriGO software (v1.2; Du et al., 2010) with MSU-RGAP 7 as the reference annotation set. Rice GO information for biological process, cellular component, and molecular function was used for gene ontology enrichment analysis. For pathway analysis of differentially expressed genes, the MapMan software (version 3.5.1; http://mapman.gabipd.org/web/guest) was used with a *P*-value cutoff of ≤0.05 and mapping files from MSU-RGAP 7.

## Results

### *OsGW2* Sequence Variation in Rice Genotypes

**Figure 1:**
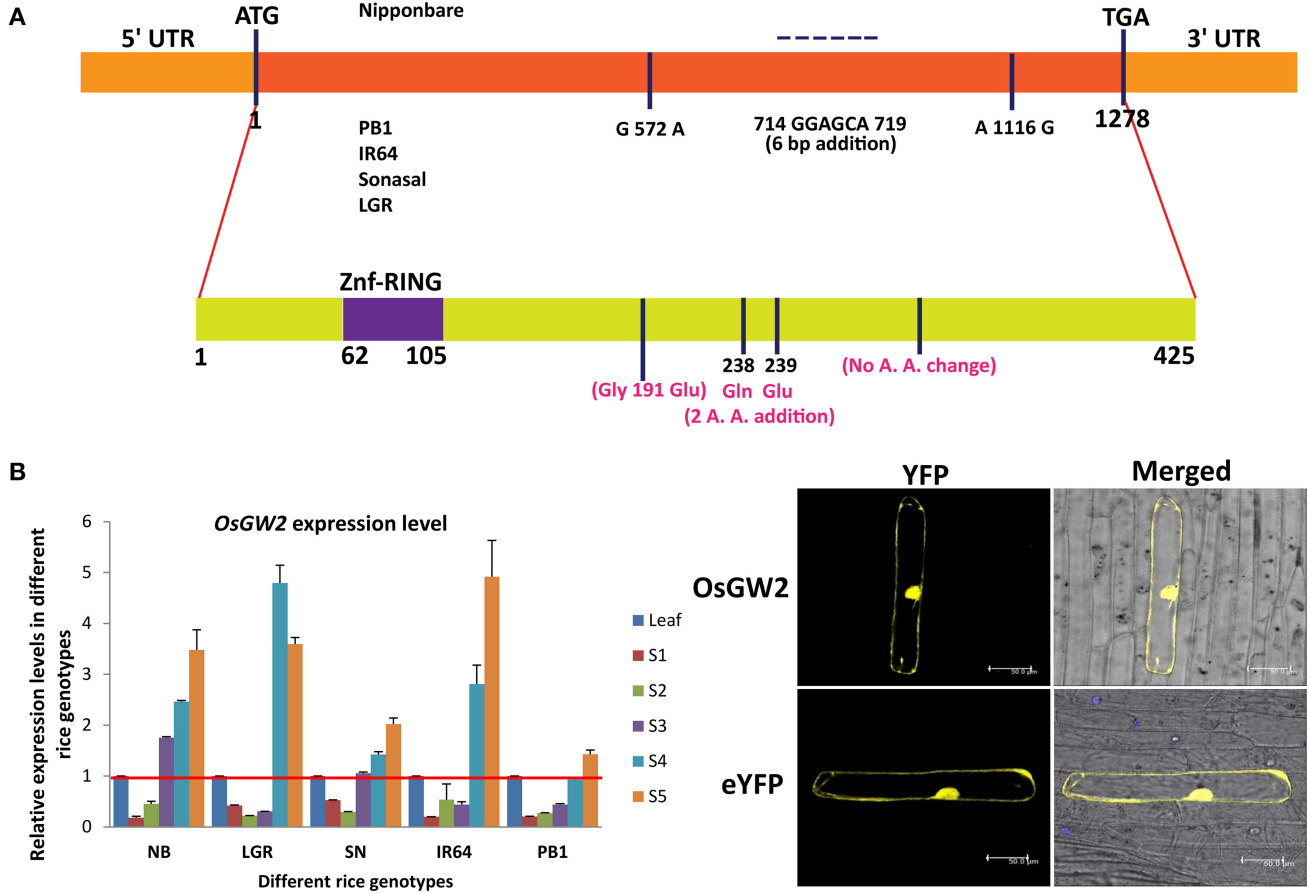
Structure, expression, and subcellular localization of OsGW2. **(A)** *OsGW2* nucleotide (upper panel) and amino acid (lower panel, marked in pink color) sequence variation in *indica* rice genotypes (IR64, PB1, Sonasal, and LGR) including substitutions and additions in comparison with the *japonica* rice genotype (Nipponbare) and protein structure variation. A. A. represents amino acid. **(B)** Expression levels of *OsGW2* in seed tissues (S1–S5) of five different rice genotypes determined by qRT-PCR. The rice *OsACTIN1* gene was used as an internal control. Data show fold changes with respect to the flag leaf of the same genotype, and bar represents mean ± SD (*n* = 3). **(C)** Subcellular localization of YFP-tagged OsGW2 in onion peel cells (top panel). Lower panel represents empty vector control. Scale bar, 50 μm.

A natural variation (single-nucleotide polymorphism) was present in large grain-sized *japonica* rice genotypes in comparison with small grain-sized genotypes which was responsible for truncation of encoded OsGW2 protein functional domain in the large grain-sized genotypes (Song et al., 2007). No such variation was observed in *indica* genotypes (Dixit et al., 2013). In our analysis, the coding region of *OsGW2* (1,278 bp) from four *indica* rice genotypes, selected on the basis of different grain sizes as extra large, large, medium, and small (LGR, PB1, IR64, and Sonasal), was sequenced and compared with *japonica* cultivar Nipponbare (Figure 1A). The coding region showed 100% similarity amongst all four *indica* rice genotypes (LGR, PB1, IR64, and Sonasal). However, the *japonica* cultivar, Nipponbare, showed variation with respect to *indica* sequence. Comparison of the nucleotide sequences of the alleles of *OsGW2* from both *indica* and *japonica* cultivars revealed three nucleotide changes, namely two single-nucleotide polymorphism (SNP) variations and one 6-bp addition in the coding region of *indica* genotypes. SNP variation (G to A) at position 572 bp resulted in an amino acid change (Gly to Glu), six base pair addition at positions 714–719 resulted in the addition of two amino acids (Gln and Glu), whereas (A–G) SNP variation at 1,116 bp did not result in any amino acid variation (Figure 1A). Despite these sequence variations, the conserved domain structure of the OsGW2 protein remained the same, and it did not show truncation as reported for *japonica* rice genotypes with increased grain size (Song et al., 2007).

### *OsGW2* Expresses Mainly in Seed Stages and Its Protein Localizes to the Nucleus and Cytoplasm

To determine the expression patterns of *OsGW2*, transcript levels were examined by qRT-PCR in S1–S5 seed developmental stages of four *indica* and one *japonica* rice genotype in comparison with their respective flag leaf. These rice genotypes show variation in grain size parameters, viz. grain width, length, and weight (Supplementary Figure 1). Previous data showed that *OsGW2* expressed constitutively in vegetative as well as reproductive tissues (Song et al., 2007). qRT-PCR data revealed that *OsGW2* expressed at lower levels in S1–S3 stages and had higher expression in S4–S5 stages of seed development, which indicates its essential role in rice seed development (Figure 1B). To study the subcellular localization of OsGW2, OsGW2 yellow fluorescent protein (YFP-OsGW2) fusion construct was designed where expression was controlled by the CaMV *35S* promoter. *In vivo* protein targeting in onion epidermal cells by bombarding with the YFP-OsGW2 fusion construct showed that it localized to both the nucleus and cytoplasm. This was observed in six independent cells (Figure 1C).

### Suppressed Expression of *OsGW2* Alters Grain Morphology

**Figure 2:**
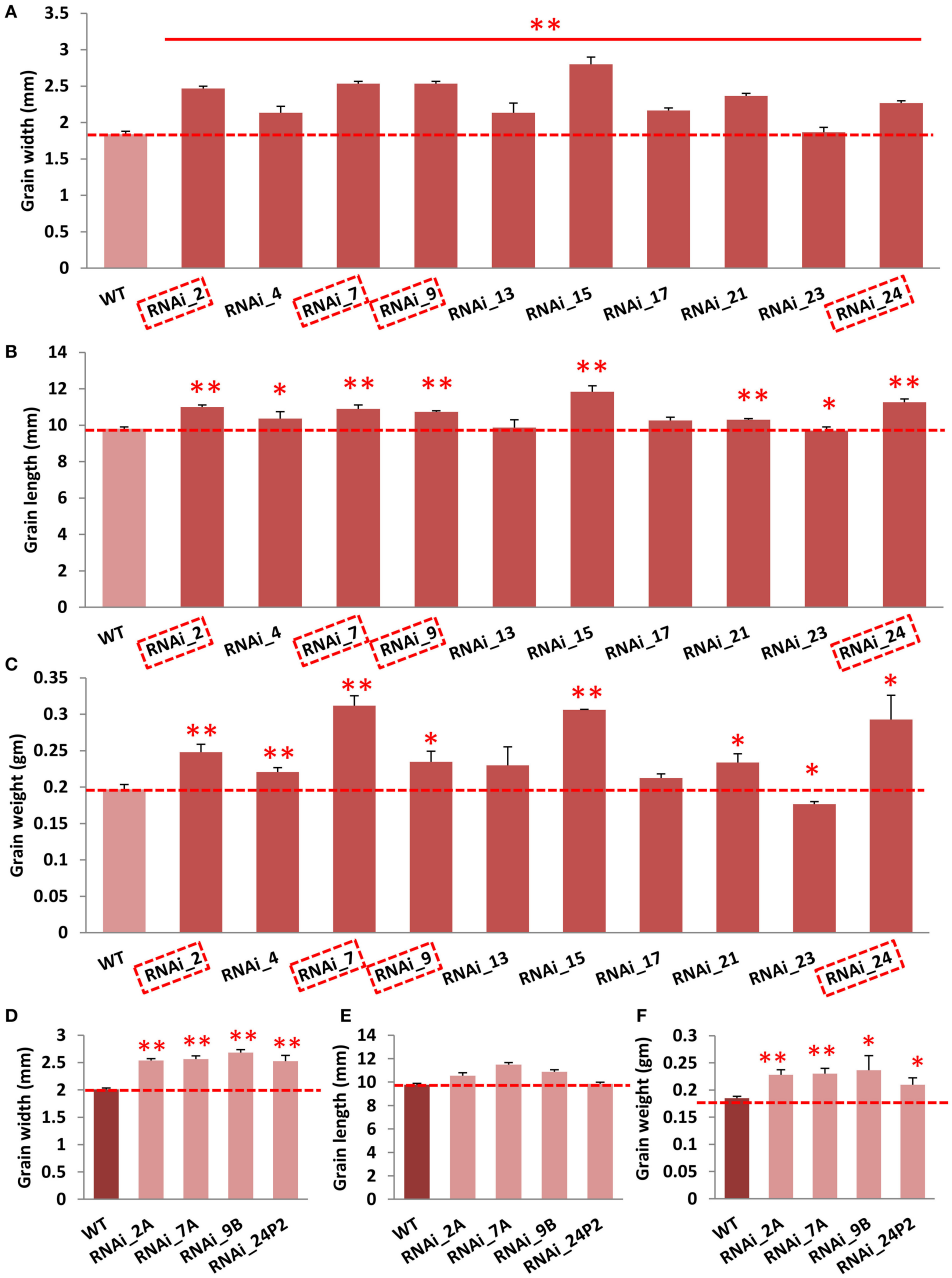
Effect of *OsGW2* knockdown on width, length, and weight of T_1_ and T_2_ rice grains. **(A–C)** Grain width, length, and 10-grain weight, respectively, of *OsGW2*_RNAi T_1_ seeds. **(D–F)** Grain width, length, and 10-grain weight, respectively, of *OsGW2*_RNAi T_2_ seeds. WT represents wild-type PB1 grains; grain length and width were measured with WINSEEDLE^TM^. RNAi_2, 4, 7, 9, 13, 15, 17, 21, 23, and 24 are 10 different transgenic lines in T_0_ generation. Dashed boxes in **(A–C)** represent selected transgenic lines for further analyses which follow 3:1 segregation in T_1_ generation. Hence, RNAi_2A, 7A, 9B, and 24P2 are four different transgenic lines in T_1_ generation, selected from T_0_ generation. Bar represents mean ± SD [*n* = 10 in **(A–E)**; *n* = 3 in **(C,F)**]. **P < 0.01; *P < 0.05, determined by Student's *t*-test.

**Figure 3:**
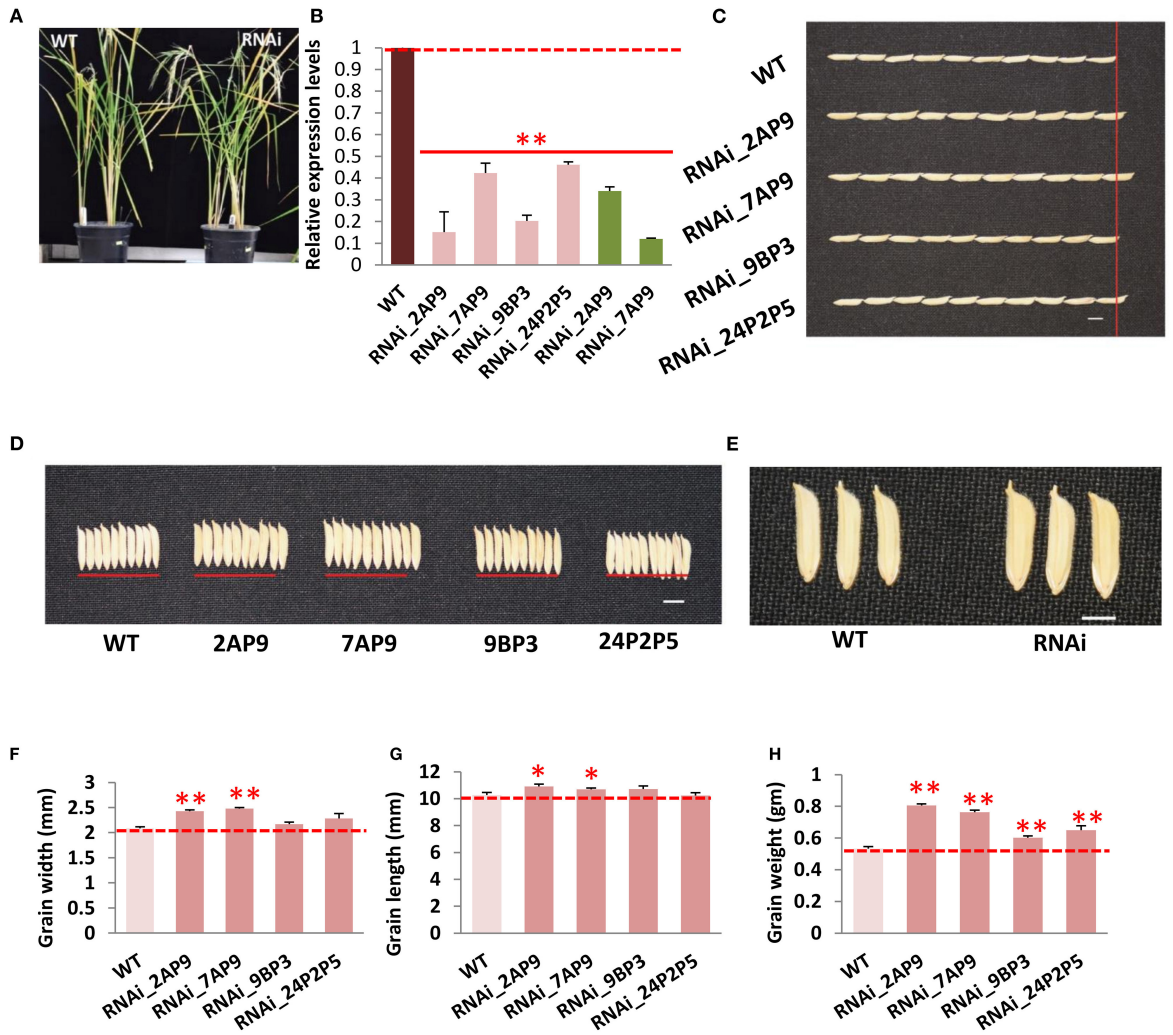
Phenotypic characterization of *OsGW2*_RNAi T_2_ generation transgenic plants. **(A)** Gross morphology of wild-type (WT) and *OsGW2*_RNAi plants at the mature stage. **(B)** Relative expression levels, by qRT-PCR, of *GW2* in *OsGW2*_RNAi plants (T_2_ flag leaf, pink bars and T_3_ grain, green bars) as compared with the respective wild-type tissues. Data show fold changes in expression level with respect to the wild-type lines; values represent mean ± SD (*n* = 3), **P < 0.01, Student's *t*-test. **(C)** Grain length of *OsGW2_*RNAi T_3_ seeds. **(D)** Grain width of *OsGW2* RNAi T_3_ seeds. **(E)** Zoomed in image of grains of WT and *OsGW2*_RNAi_7AP9 T_3_ grains. Bar = 1 cm. Comparison of **(F)** grain width, **(G)** grain length, and **(H)** 30-grain weight of *OsGW2*_RNAi T_3_ seeds with wild-type PB1 seeds. Error bar represents mean ± SD [*n* = 100*3 in **(F,G)**, *n* = 3 in **(H)**]. **P ≤ 0.01, and *P ≤ 0.05, calculated by Student's *t*-test. RNAi (2AP9, 7AP9, 9BP3, 24P2P5) are four different lines in T_2_ generation.

To functionally validate *OsGW2*, we generated *OsGW2* RNA interference (RNAi) lines in *indica* PB1 (IET-10364) background, and a 510-bp unique region was targeted to suppress the expression of *OsGW2*. A total of 10 *OsGW2*_RNAi transgenic lines (RNAi_2, 4, 7, 9, 13, 15, 17, 21, 23, 24) were obtained through tissue culture. Each plant of all these transgenic lines in T_0_ generation was found positive by PCR amplification of hygromycin resistance gene (*Hygromycin phosphotransferase II*) resulting in an amplicon of 850 bp (Supplementary Figure 2). Vegetative parameters (plant height, flag leaf length, flag leaf width, number of tillers per plant) did not show any morphological difference between RNAi and wild-type PB1 plants. However, transgenic plants in T_0_ generation showed significantly higher grain width (23–38%), grain length (5–15%), and grain weight (19–58%) when compared with the wild-type lines (Figures 2A–C; Supplementary Figure 2). In T_1_ generation, four lines (RNAi 2A, 7A, 9B, and 24P2) were selected out of 10 lines which showed 3:1 segregation ratio (Supplementary Table 2). These *OsGW2* RNAi plants had significantly higher grain width, length, and weight in comparison with PB1 seeds, in T_1_ generation (Figures 2D–F). Other phenotypic parameters did not show significant difference in comparison with wild-type plants as before. The four lines were taken forward, and in T_2_ generation, suppressed expression of *OsGW2* (RNAi 2AP9, 7AP9, 9BP3, and 24P2P5) was screened and used for further analysis. Two lines 2AP9 and 7AP9 had homozygous plants. Positive *OsGW2*_RNAi transgenic plants along with the wild-type lines were grown and analyzed (Figure 3A; Supplementary Figure 3). Expression levels were significantly reduced (53.88–84.84%) in the third leaf (as the gene expresses ubiquitously) of *OsGW2*_RNAi T_2_ transgenic plants. T_3_ transgenic seeds from S4 stage of the two homozygous lines also showed a significant decrease (66–88%) in the expression of *GW2* (Figure 3B). The grains of T_2_ generation plants were significantly larger than those of the wild-type lines (Figure 3E), showing increased grain width (4.0–18.98%) and grain length (4.09–6.19%) (Figures 3C,D,F,G). GW2 is known to affect seed width in *japonica* rice (Song et al., 2007). It has also been shown to affect width and, hence, weight in wheat (Sestili et al., 2019). In our experiments, T_1_ seeds showed a significant increase in length. T_2_ seeds did show an increase in length but it was not significant (Figure 2E). Again, T_3_ seeds from two homozygous lines (Figure 3G) showed a significant increase in length. This may be possible because the *t*-test was performed on 10 T_1_ seeds from one plant, in triplicates. Further, 10 T_2_ seeds were taken from three plants and 100 T_3_ seeds were taken from three plants to calculate seed parameters. All T_1_ seeds generated by each plant and T_3_ seeds from two homozygous plants showed a significant increase in seed length, probably because of less variation in the sample taken. Furthermore, the 30-grain weight at the mature stage of *OsGW2*_RNAi T_3_ seeds was 12.94–42.96% higher than that of the wild-type grains (Figure 3H). These results indicated that reduced expression of *OsGW2* could significantly increase grain width and weight even in *indica* rice, where a natural polymorphism was absent.

### *OsGW2* Regulates Rice Grain Size by Influencing Cell Division and Expansion

**Figure 4:**
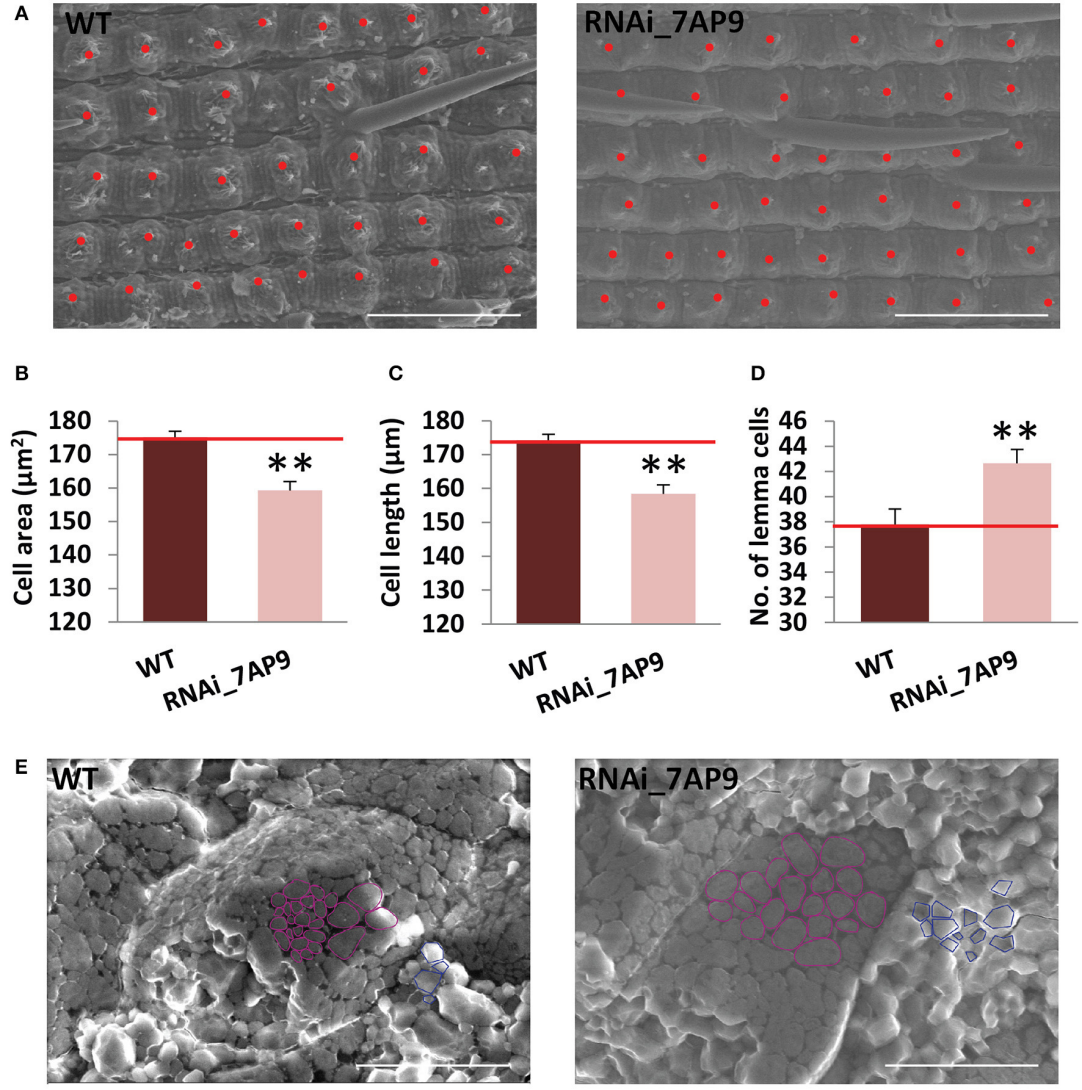
*OsGW2* influences cell proliferation and expansion in T_3_ spikelet hulls. **(A)** SEM photographs of the outer surface of lemma from wild-type and *OsGW2*_RNAi_7AP9 T_3_ seeds. Each red dot represents a cell. **(B–D)** Cell area, cell length, and number of cells on the epidermis of lemma surface as seen in the area under the microscope. Bar represents mean ± SD; *n* = 3. **P ≤ 0.01, calculated by Student's *t*-test. **(E)** Scanning electron microscopy (SEM) analysis of starch granules of endosperm in the wild-type and *OsGW2*_RNAi_7AP9 T_3_ seeds at the mature stage. As a representation, spherical granules are outlined in magenta, while the polyhedron ones are by a blue outline. WT represents wild-type; RNAi_7AP9 represents the knockdown line of *OsGW2*. Scale bar in **(A)** and **(E)** = 20 μm.

Spikelet hull size is delimiting to the final grain size. Since cell proliferation and/or cell expansion is responsible for alteration in grain size, the cell number of the *OsGW2*_RNAi_7AP9 line was calculated in the central part of the lemma epidermis using scanning electron microscopy images (Figure 4A). The cell length and cell area of the outer lemma epidermis of the wild-type and *OsGW2*_RNAi_7AP9 T_3_ grains were compared by SEM (Figures 4B,C). The images revealed that total lemma cell number in *OsGW2*_RNAi was increased by 12.94% compared with that of the wild-type lines (Figure 4D), resulting from a significant decrease in cell length and cell area. This suggested that decreased expression level of *OsGW2* contributes to higher grain width by affecting both cell division and cell expansion to regulate the number and size of cells of the spikelet hull during the process of development. To check whether or not the suppressed expression of *OsGW2* influences the structure of starch granules in the endosperm, we observed seed cross-sections by a scanning electron microscope. In the WT endosperm, starch granules were tightly packed. The spherical starch granules (Dong et al., 2015) were more in comparison with the polyhedron-shaped ones. However, in the *OsGW2*_RNAi_7AP9 line, starch granules were loosely packed and were larger in size, and the distribution of spherical and polyhedron granules was similar (Figure 4E). Thus, suppression of *OsGW2* additionally resulted in modifications in starch granule size.

### Transcriptome Analysis

**Figure 5:**
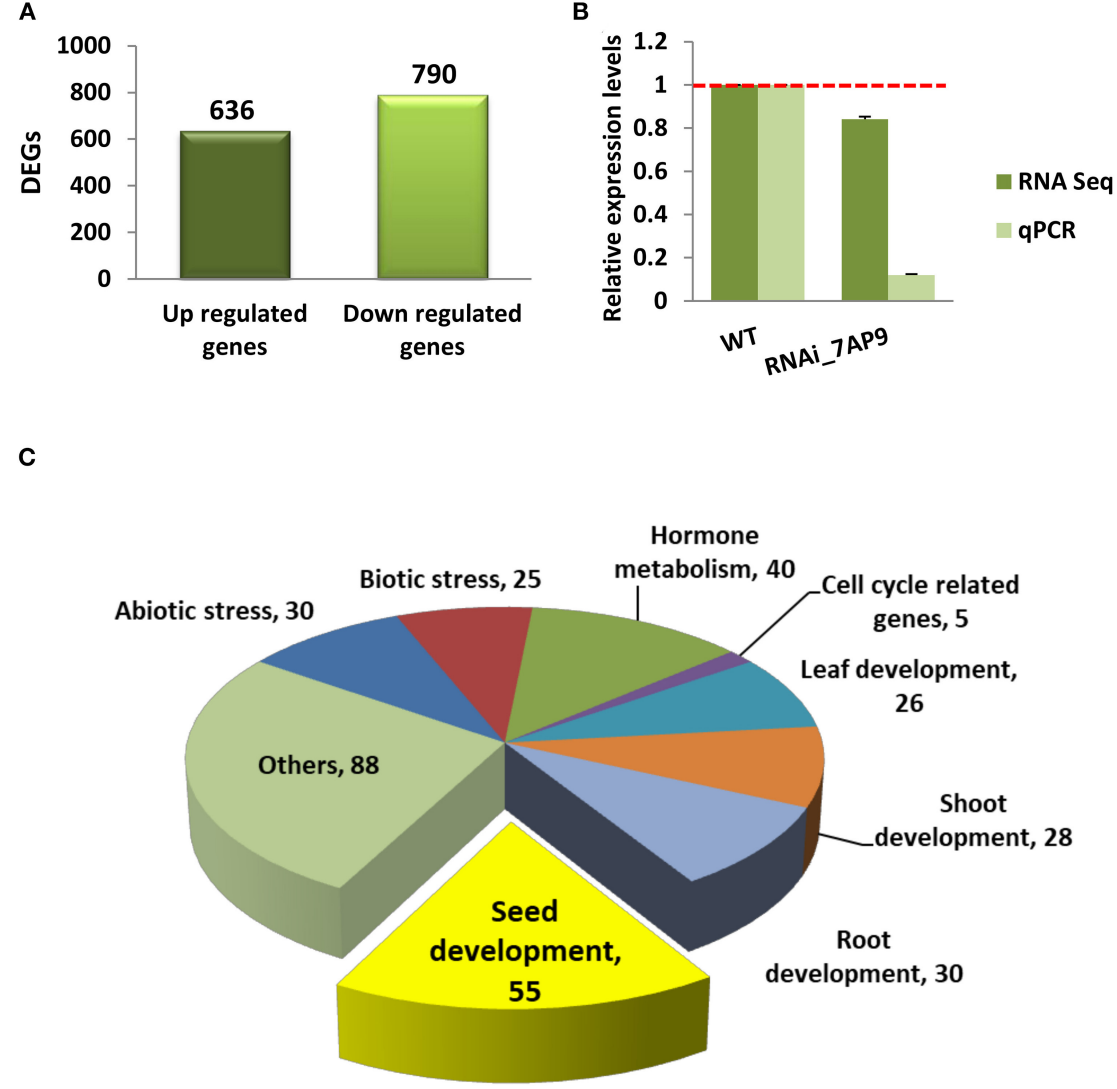
Transcriptome analysis of *OsGW2*_RNAi T_3_ generation S4 stage seeds. **(A)** The number of up- and downregulated DEGs between WT and *OsGW2*_RNAi_7AP9 seeds is represented as bars. The DEGs with log_2_ fold change ≥1.5 (upregulated genes) and ≤-1.5 (downregulated genes) with a *P-*value cutoff of ≤0.05 were considered as significant DEGs. **(B)** qRT-PCR validation of RNA-seq-based expression levels of *OsGW2*. qRT-PCR values are the same as in Figure 3B. Data are mean ± SD for two biological replicates. **(C)** Categorization of functionally characterized DEGs with their overlapping functional terms through RICENCODE. Yellow color in the pie chart represents DEGs specifically involved in seed development. Colors in the pie chart represent different plant development and metabolism-related processes.

The transcriptome of the developing seed was compared at S4 stage between WT and *OsGW2*_RNAi_7AP9 (a homozygous line) to elucidate genes and pathways which could be altering the grain size. Two biological replicates of each sample were used for sequencing and four libraries were generated in total. Each library had more than 50–78 million clean reads after quality control and 89–93% reads mapped uniquely to the rice reference sequences (Supplementary Table 3). Pearson correlation coefficient (*R*^2^) was calculated between biological replicates. Correlation was higher (0.98) within the biological replicates than that between the WT and *OsGW2*_RNAi_7AP9 line. Published data shows that in rice, using RNA sequencing data from two biological replicates, ~22–61 million clean reads have been generated and 62–92% of these reads map to the reference genome (Ding et al., 2017; Yuenyong et al., 2018; Jung et al., 2019; Zha et al., 2019). The transcripts with log_2_ fold change ≥1.5 (upregulated genes) and ≤(−1.5) (downregulated genes) with a *P-*value cutoff of ≤0.05, in *OsGW2*_RNAi_7AP9 transgenic line in comparison with WT, were considered as DEGs. Thus, we could identify 1,426 DEGs, of which 636 (44.60%) DEGs had higher expression in the *OsGW2*_RNAi_7AP9 line compared with the WT (upregulated genes), while 790 (55.40%) DEGs showed lower expression in the *OsGW2*_RNAi_7AP9 line compared with the WT (downregulated genes) (Figure 5A; Supplementary Table 4). The expression data obtained through RNA sequencing was validated by comparing the expression of *OsGW2* in *RNAi_7AP9* T_3_ seeds generated by qRT-PCR (Figure 3B). The expression patterns were found consistent for *OsGW2* in both analyses (Figure 5B).

All identified DEGs (1,426 in total) in *OsGW2*_RNAi_7AP9 plants were analyzed by comparison with functionally characterized rice genes from the RICENCODE database. A total of 115 genes were well characterized with specific function (Supplementary Table 5). These genes were involved in the regulation of development of the seed, leaf, root, and shoot, apart from the genes related to the cell cycle, hormone signaling, and biotic and abiotic stress tolerance. Overlapping functions of these 115 characterized genes were represented a total of 327 times with respect to different functional terms (leaf, root, shoot, flower, and seed development, etc.). Of these, 55 genes were known to be involved in seed development in rice (Figure 5C, Supplementary Table 6).

**Figure 6:**
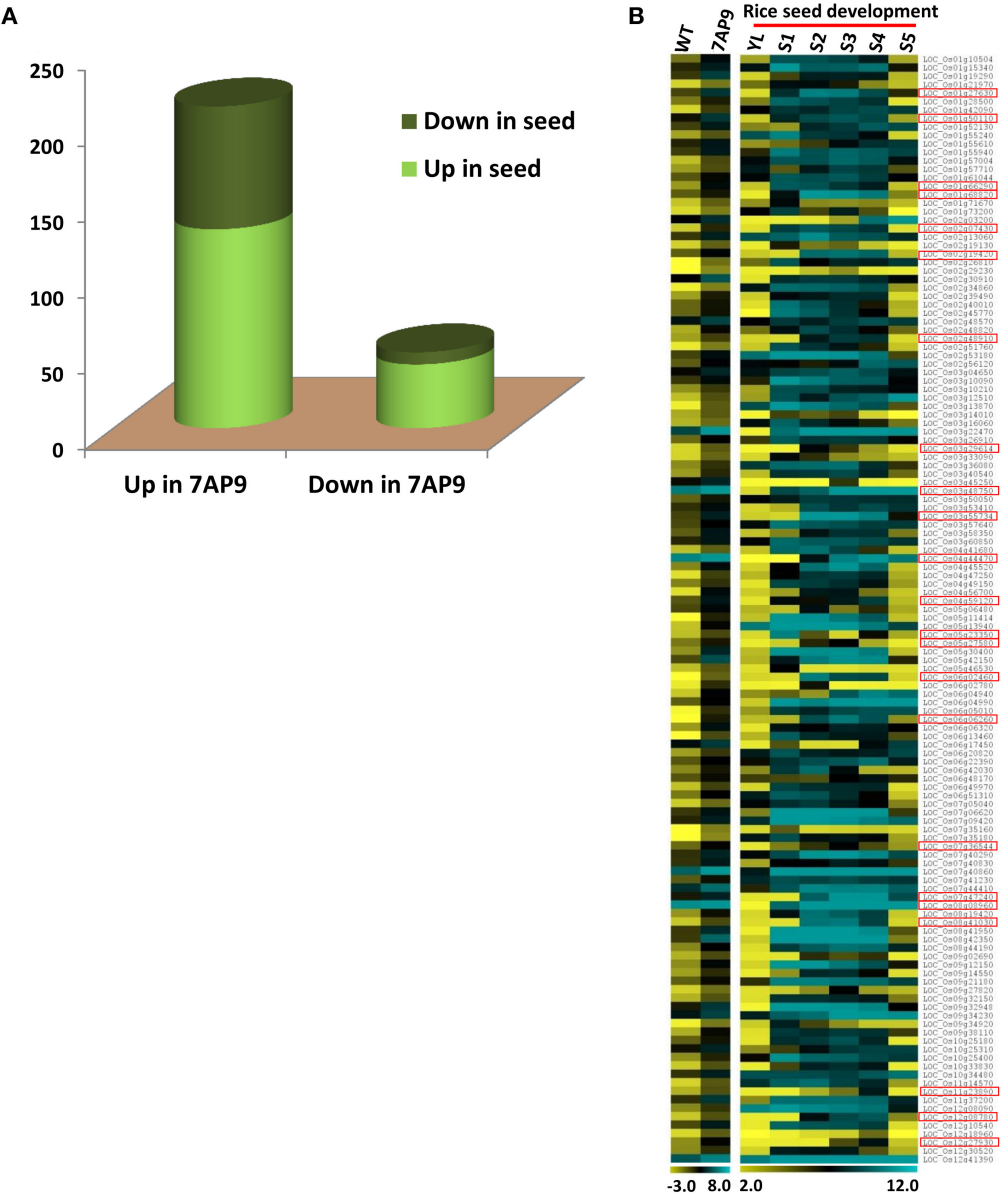
Comparison of transcriptomes of *OsGW2*_RNAi seed and five rice seed development stages. **(A)** Graph represents DEGs in both IR64 seed development stages and in *OsGW2*_RNAi_7AP9 seeds. The first and second bars represent gene numbers up- and downregulated, respectively, in *OsGW2*_RNAi_7AP9 seeds. Gene numbers up- and downregulated in rice seed are marked in light and dark green, respectively. **(B)** Heat map shows log_2_ expression values of genes upregulated in both *OsGW2*_RNAi_7AP9 seeds and in five stages of rice seed development. Upregulated seed-specific genes have been demarcated with red boxes in the figure. Color bar below the heat map shows the range of log_2_ GCRMA or FPKM values, for microarray and RNA-seq, respectively. YL represents Y-leaf and S1-S5 represent five stages of rice seed development.

To gain insight into the function of *OsGW2* in seeds, DEGs in *OsGW2*_RNAi_7AP9 T_3_ seeds were compared with previously generated DEGs in five seed developmental stages (S1–S5) in the rice genotype IR64 (Sharma et al., 2012). The same analysis has also been successfully done for rice transgenic plants with altered expression of another seed-specific gene *ONAC025*. DEGs common to both transgenic plant and seed are the ones downstream to the transgene (Mathew et al., 2020). This analysis unveiled a correlation of DEGs in the *OsGW2*_RNAi_7AP9 transgenic line with seed-related genes. Of the 636 genes upregulated as a result of *OsGW2* knockdown, 262 genes were also differentially expressed in the different seed developmental stages in comparison with the Y-leaf stage (Figure 6A). Of these, 131 genes were upregulated in both *OsGW2*_RNAi_7AP9 plants and S1–S5 seed developmental stages, of which 23 were seed-specific (Figure 6B). The upregulated seed-specific genes in *OsGW2*_RNAi_7AP9 plants were involved in various seed development and related processes/pathways. One such seed-specific transcription factor, *OsMADS29*, has been shown to affect many processes. Knockdown and overexpression of *OsMADS29* revealed its function in hormone homeostasis, starch biosynthesis in the endosperm, and embryo development (Yang et al., 2012; Nayar et al., 2013). Also, 81 genes showed downregulation in various seed stages compared with the Y-leaf stage (Supplementary Figure 6A). Similarly, among the 50 downregulated genes in *OsGW2*_RNAi_7AP9 plants, 42 showed upregulation in the seed stages and eight of them showed downregulation in the seed stages compared with the Y-leaf stage (Figure 6A; Supplementary Figures 6B,C).

**Figure 7:**
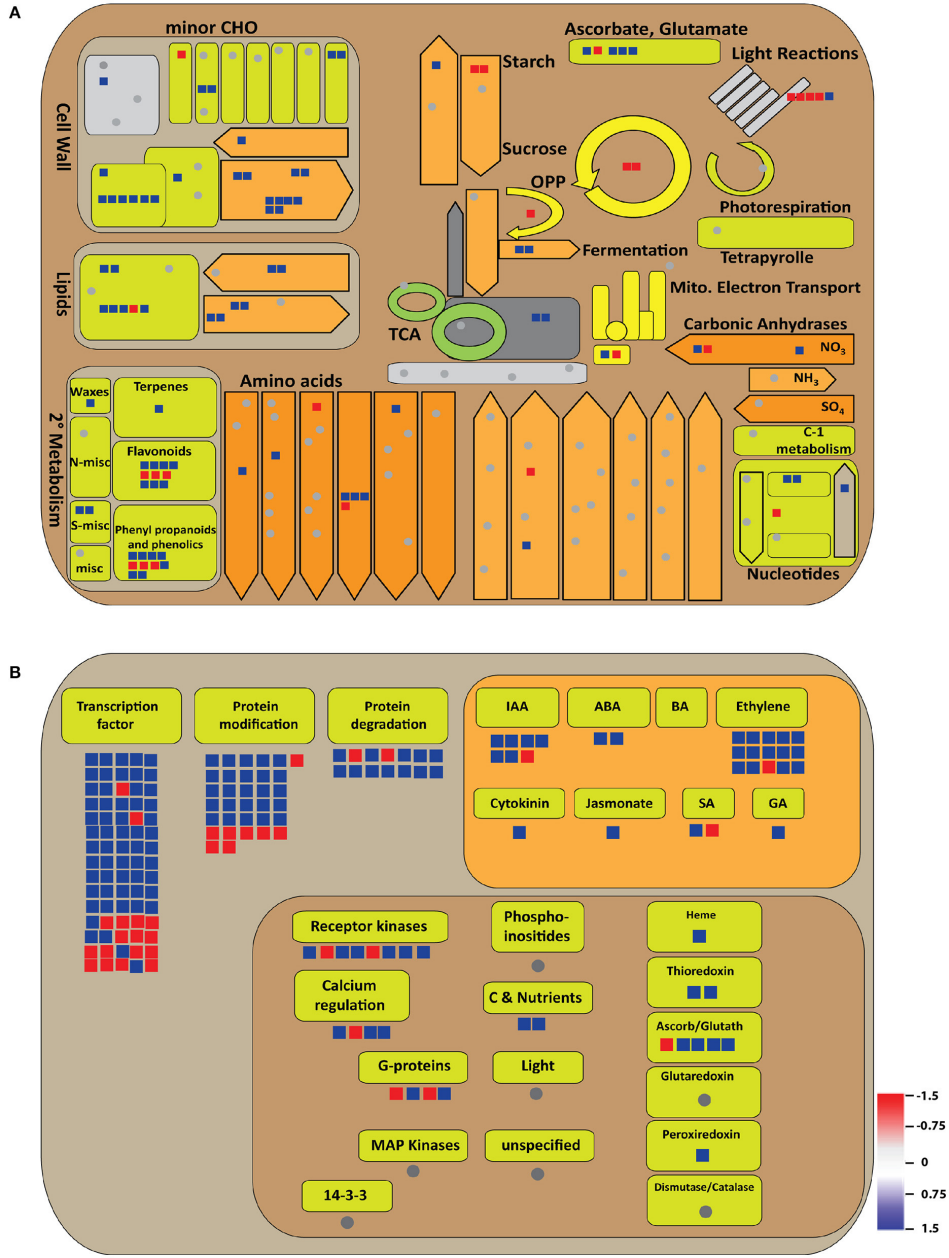
Illustration of metabolic processes involving significant DEGs (log_2_ fold change ≥1.5 or ≤−1.5) as visualized by the MapMan tool. **(A)** Metabolism and **(B)** regulation overview have been shown. Blue and red colors signify up- and downregulated genes, respectively, as shown in the color bar in **(B)**.

Furthermore, the total DEGs were analyzed for their roles in metabolic pathways by MapMan. There was enrichment of secondary metabolites, starch and sucrose biosynthesis, and lipid metabolism, besides amino acid metabolism and cell wall biosynthetic pathways in the *OsGW2*_RNAi_7AP9 line (Figure 7A). A parallel level of pathway enrichment was also observed by KEGG. KEGG analysis of all DEGs revealed that photosynthesis, sucrose, and starch metabolism DEGs were mostly upregulated (Supplementary Figure 4). We found that genes involved in signaling pathways, including components of ABA, cytokinin, ethylene, GA, IAA, jasmonic acid, and transcription regulatory elements (including TFs), were enriched. Similarly, genes involved in protein modification and protein degradation were found to be enriched in *OsGW2*_RNAi_7AP9 (Figure 7B).

## Discussion

*GRAIN WIDTH 2* codes for an E3 ubiquitin ligase. A natural variation in *GW2* causes truncation of the protein-interacting domain in a long grain *japonica* rice cultivar, WY3, resulting in increased grain width and weight (Song et al., 2007). Recent steps toward the identification of the molecular mechanism of GW2 show that it interacts with EXPLA1, a cell wall-loosening protein (Choi et al., 2018), and chitinase 14 (CHT14) and phosphoglycerate kinase (PGK) which are involved in carbohydrate metabolism (Lee et al., 2018). Oochikara (a natural *japonica* rice gw2 mutant) has loosely packed spherical starch granules (Choi et al., 2018), also observed in knockdown *indica* rice in our experiments (Figure 4E). Since *indica* rice lacks the SNP leading to a stop codon (Dixit et al., 2013), the role of such a *GW2* allele has hitherto not been explored in these varieties. This is the first report of functional relevance of *GW2* in *indica* rice, which shows that its downregulation promotes seed width, length and weight, even in *indica* rice, and elucidates the genes downstream to GW2 responsible for this phenotype.

In the present study, four *indica* rice genotypes, with variation in seed size, lacked the natural variation of *japonica* genotypes, though *GW2* expressed in all. In order to test if the GW2 protein has a functional role in *indica* rice, we generated a knockdown of *OsGW2* in *indica* rice. The aim was to examine if a knockdown would generate a phenotype similar to a knockout phenotype observed in *japonica* genotypes. A similar RNAi-based knockdown approach has been adopted to explore the *GW2* function in wheat which resulted in higher grain width and starch content (Sestili et al., 2019). While knockout (KO) is increasingly being used to study gene functions, the methodology has certain limitations, such as the unavailability of PAM sites, off-target effects, and most importantly a “fitness cost” (Ahmad et al., 2018). Partial knockdown of genes allows us to study the function of essential genes, while complete knockout of genes may be lethal to the plants (Boettcher and McManus, 2017; Moreira et al., 2020). Hence, RNAi-mediated knockdown of genes has its own utility. It has also been suggested to first characterize a gene by RNAi methodology and, subsequently, edit it by the CRISPR/Cas technique (Ansari et al., 2020). We did not generate KO in this attempt as the gene expresses ubiquitously, and a KO may hamper plant growth.

Grain weight, a key determinant of grain yield in rice, is positively associated with grain size parameters (grain length, width, and thickness; Shi et al., 2019). Majority of the genes (*GW5, qGL3, UBP15/LG1, GL4, SPL16*, etc.) impact grain size by affecting the rate of cell division/proliferation. Few genes (*SPL13, KNAT7*) were reported to control grain size by influencing cell expansion (Weng et al., 2008; Qi et al., 2012; Zhang et al., 2012; Wang et al., 2015, 2019; Si et al., 2016; Liu et al., 2017; Wu et al., 2017; Gao et al., 2019; Shi et al., 2019). In the last decade, some genes were reported that regulate grain size by controlling both cell proliferation as well as expansion (*GS2, GS5*; Li et al., 2011; Duan et al., 2015; Xu et al., 2015). In our study, SEM analysis of the central portion of the hull of mature grains of the knockdown line showed that an increase in grain size resulted from increased cell number. Previously, in *Arabidopsis*, it was observed that E3 ubiquitin ligase, *DA2*, and *EOD1* also regulate grain size by affecting cell proliferation (Li et al., 2008; Xia et al., 2013). GW2 controls grain size in rice in a similar way as DA2 and EOD1 in *Arabidopsis*. Downregulation of *OsGW2* led to a wider and larger grain. Collectively, *OsGW2* negatively regulates grain size by affecting cell proliferation in the spikelet hull even in *indica* rice.

In dicot plants, endosperm disappears with embryo maturation, whereas in monocots, the endosperm covers the major proportion of the seed after maturation and stores starch in amyloplasts (Olsen et al., 1999). Sugars translocate to the endosperm from vegetative tissues and are converted to amylose and amylopectin at the time of grain filling and stored in the form of starch granules in amyloplasts (Nayar et al., 2013; Sonnewald and Kossmann, 2013). The starch granules in the endosperm of the *OsGW2* knockdown lines were larger and more loosely packed. *OsGW2* knockdown affected the size, shape, and number of starch granules. Transcriptome data suggested that transcript levels of starch metabolism/synthesis-related genes (*OsIAA9, OsMADS6, OsMADS29*) are upregulated in the silenced *OsGW2*_RNAi plants (Supplementary Table 6).

Hormone signal transduction, cell cycle, and sucrose metabolism-related genes are well known to be involved in grain size regulation through various metabolic processes (Li and Li, 2016; Lee et al., 2017). In our transcriptome study, KEGG, MapMan, and GO analyses revealed that DEGs that were significantly enriched pertained to biological processes (cellular process, primary and secondary metabolic processes), metabolism (carbohydrate, lipid, amino acid, and nucleotide), hormone signal transduction, starch metabolism, stress response, photosynthesis, etc. (Figure 7; Supplementary Figures 4, 5). Plant hormones are important grain size regulators. Auxins, cytokinins, and brassinosteroids play an important role in determining rice grain size. Auxin-responsive genes (*OsARF4, Gnp4*/*LAX2, BG1, qTGW3, TGW6*), a cytokinin-related gene (*OsCKX2*/*Gn1a*), and brassinosteroid-related genes (*GS5, qGL3, GS2*/*OsGRF4*) regulate grain size by being involved in signaling, biosynthesis, and transport (Azizi et al., 2019; Li and Li, 2019). In our transcriptome data, majority of auxin-responsive genes, including *OsMGH3, OsMADS29, OsRAA1*, and *OsIAA9*, showed upregulation in *OsGW2* knockdown plants (Supplementary Table 6). In addition to this, cytokinin- and brassinosteroid-related genes were also upregulated in the *OsGW2*_RNAi_7AP9 line. These results provide evidence for hormone-responsive genes being important regulators of grain size in rice. Several genes (*GS3, GS5, GW5, qGL3, TGW6, GIF1, GW8*, etc.) are preferentially or specifically expressed in seed tissues and regulate grain size in rice by being part of various pathways like the ubiquitin–proteasome pathway, G-protein signaling, plant hormone signaling, mitogen-activated protein kinase (MAPK) signaling, and some transcription factors (Zuo and Li, 2014). Upregulation of starch metabolism and cell cycle-related genes (*OsMADS6, OsMADS29, OsYUC9*, and *OsKRP3*) in *OsGW2* knockdown plants also indicates the pathways through which GW2 regulates seed development in rice. The DEGs generated by transcriptome analysis will in all probability be downstream of OsGW2 substrates, which regulate grain size in rice. We can speculate that upregulated genes in *OsGW2*_RNAi plants that are also seed-specific might be involved in direct grain size regulation.

## Conclusion

Here, we described some important facets of *OsGW2*-mediated regulation of grain width and weight in *indica* rice. Both cell division and expansion in the hull of spikelet are responsible for higher grain width as well as weight in *OsGW2* knockdown plants in *indica* rice. Additionally, *OsGW2* also affects starch granule morphology and their packing. We further conducted RNA sequencing to elucidate the active molecular mechanisms in *OsGW2* knockdown plants and identified enriched DEGs involved in hormone signal transduction and starch metabolism. Transcriptome analyses also suggested that *OsGW2* plays a crucial role in the regulation of cellular and metabolic processes relevant to grain size regulation.

## Data Availability Statement

RNA-seq was performed for one wild type line and one transgenic line (RNAi_7AP9), in biological replicates, using Illumina® HiseqTM. RNA sample preparation kit v.2 (Illumina; USA). The sequences generated were submitted to NCBI SRA database (http://www.ncbi.nlm.nih.gov/sra/PRJNA659415) with SRA accession number PRJNA659415. Biosample accession numbers for individual samples are SAMN15905438_WT_Rep1, SAMN15905439_WT_Rep2, SAMN15905440_7AP9_Rep1, SAMN15905441_7AP9_Rep2.

## Author Contributions

GP made the constructs, and RR and GP generated the rice transgenic plants. AV conducted all the experiments and analyzed the data. AKT and PA supervised the study and guided the experimental design and data analysis. AV and PA wrote the manuscript. All authors read/corrected the manuscript and approved it.

## Conflict of Interest

The authors declare that the research was conducted in the absence of any commercial or financial relationships that could be construed as a potential conflict of interest.

## Supplementary Material

Supplementary Figure 1 Comparison of grain parameters in five different rice genotypes. **(A)** Grain width, **(B)** grain length, and **(C)** grain weight. Error bar represents mean ± SD; *n* = 100. Grain width and length were measured with WINSEEDLE^TM^. Nipponbare, LGR, Sonasal, IR64, and PB1 represent different rice genotypes.

Supplementary Figure 2 Screening of *OsGW2*_RNAi transgenic plants and effect of GW2 on grain size parameters. **(A)** Screening of *OsGW2_*RNAi T0 transgenic plants by PCR amplification of hygromycin resistance gene (*Hygromycin phosphotransferase II*). L represents Gene Ruler 100 bp DNA Ladder Mix. 1–35 are different transgenic lines in T0 generation. Ten positive T0 plants marked in red (2, 4, 7, 9, 13, 15, 17, 21, 23, 24) were grown further and analyzed. Effect of *GW2* on grain size parameters; grain length **(B)** and width **(C)** of *OsGW2_*RNAi T1 seeds and; grain length **(D)** and width **(E)** of *GW2* RNAi T2 seeds. In T1 generation four lines (RNAi_2A, 7A, 9B, and 24P2) were selected out of ten lines which showed 3:1 segregation ratio. Bar = 1 cm; WT represents wild-type PB-1 grains; grain length and width measured with 30 cm scale by aligning 10 seeds of each line length wise and breadth wise. Red line in **(B–E)** represents WT measurements for comparison.

Supplementary Figure 3 Screening of *OsGW2_RNAi* plants in T2 generation by PCR amplification of hygromycin resistance gene (*Hygromycin phosphotransferase II*). L represents Gene Ruler 1 kb DNA Ladder Mix. RNAi (2AP3, 2AP9, 7AP8, 7AP9, 9BP3, 9BP4, 24P2P5, 24P2P6) are different lines in T2 generation. N and P represent negative and positive control, respectively.

Supplementary Figure 4 Kyoto Encyclopedia of Genes and Genomes (KEGG) pathway enrichment of DEGs. KEGG pathway analysis of **(A)** up-regulated and **(B)** down-regulated genes. The KEGG pathways with FDR value ≤0.05 were significantly enriched. Numbers represent DEGs enriched in each pathway.

Supplementary Figure 5 GO term enrichment analysis through AgriGO of DEGs in *OsGW2*_RNAi transgenic plants. The terms enriched under **(A)** biological process, **(B)** cellular component, and **(C)** molecular function have been shown. Each box contains GO terms, GO IDs, and statistical information. GO terms with FDR value ≤0.05 show significant enrichment. Yellow, orange, and red colors represent significant GO terms and white color represents non-significant terms as shown in color legend in **(B)**. Arrows represent relationship between GO terms as shown in **(B)**.

Supplementary Figure 6 Heat maps showing comparison of *OsGW2*_RNAi_7AP9 seeds RNAseq data with rice seed development microarray data. **(A)** DEGs down-regulated in IR64 seeds (S1–S5 stages) and up-regulated in *OsGW2*_RNAi_7AP9 transgenic seeds. **(B)** DEGs up-regulated in IR64 seeds and down-regulated in *OsGW2*_RNAi_7AP9 transgenic seeds. **(C)** DEGs down-regulated in both IR64 seeds as well as *OsGW2*_RNAi_7AP9 transgenic seeds. S1–S5 represent different seed developmental stages, YL represents Y leaf. WT and 7AP9 represent wild-type and *OsGW2*_RNAi transgenic line, respectively. The DEGs with log_2_ fold change ≥1.5 (up-regulated genes) and ≤−1.5 (down-regulated genes) with *p-*value cut off of ≤0.05 were considered as significant DEGs.
