# Supplementary material for: Silencing of an Ubiquitin Ligase Increases Grain Width and Weight in indica Rice
Source: Front Genet. 2021 Jan 12;11:600378. doi: 10.3389/fgene.2020.600378 (PMC7835794; doi:10.3389/fgene.2020.600378)

**
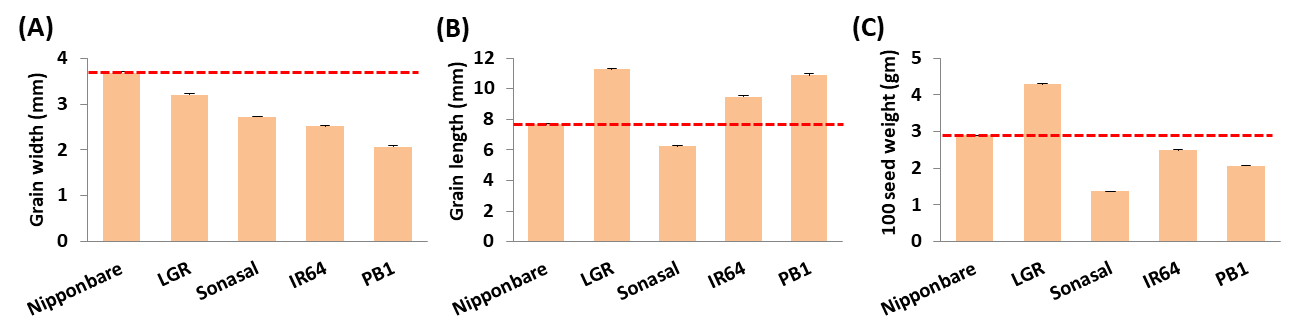
**

**Figure S1.**

**
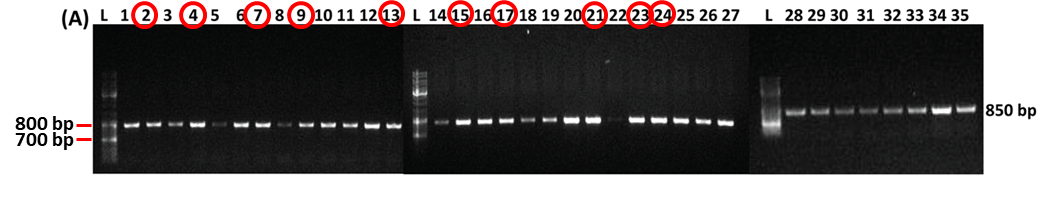
**

**Figure S2.**


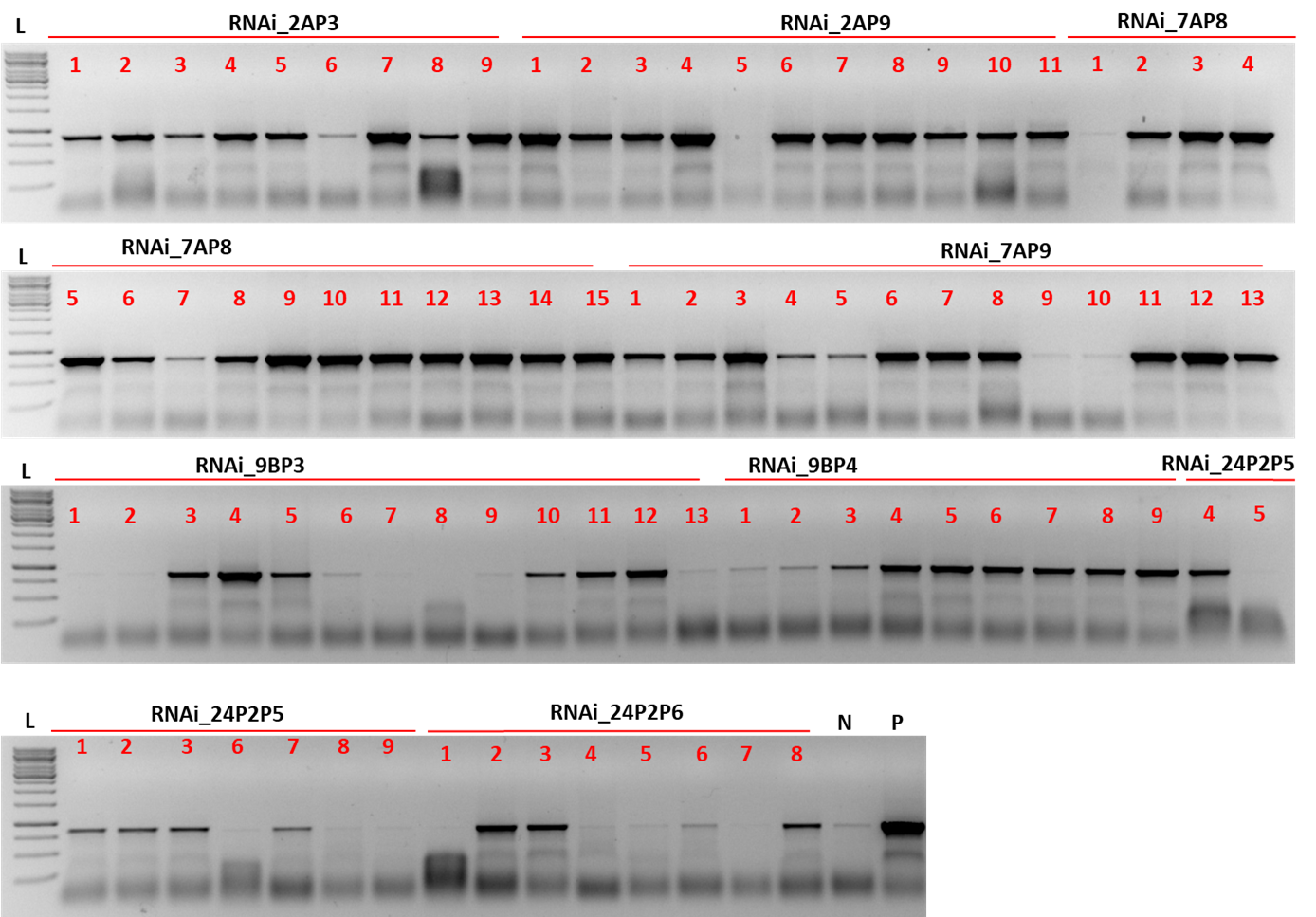


**Figure S3.**


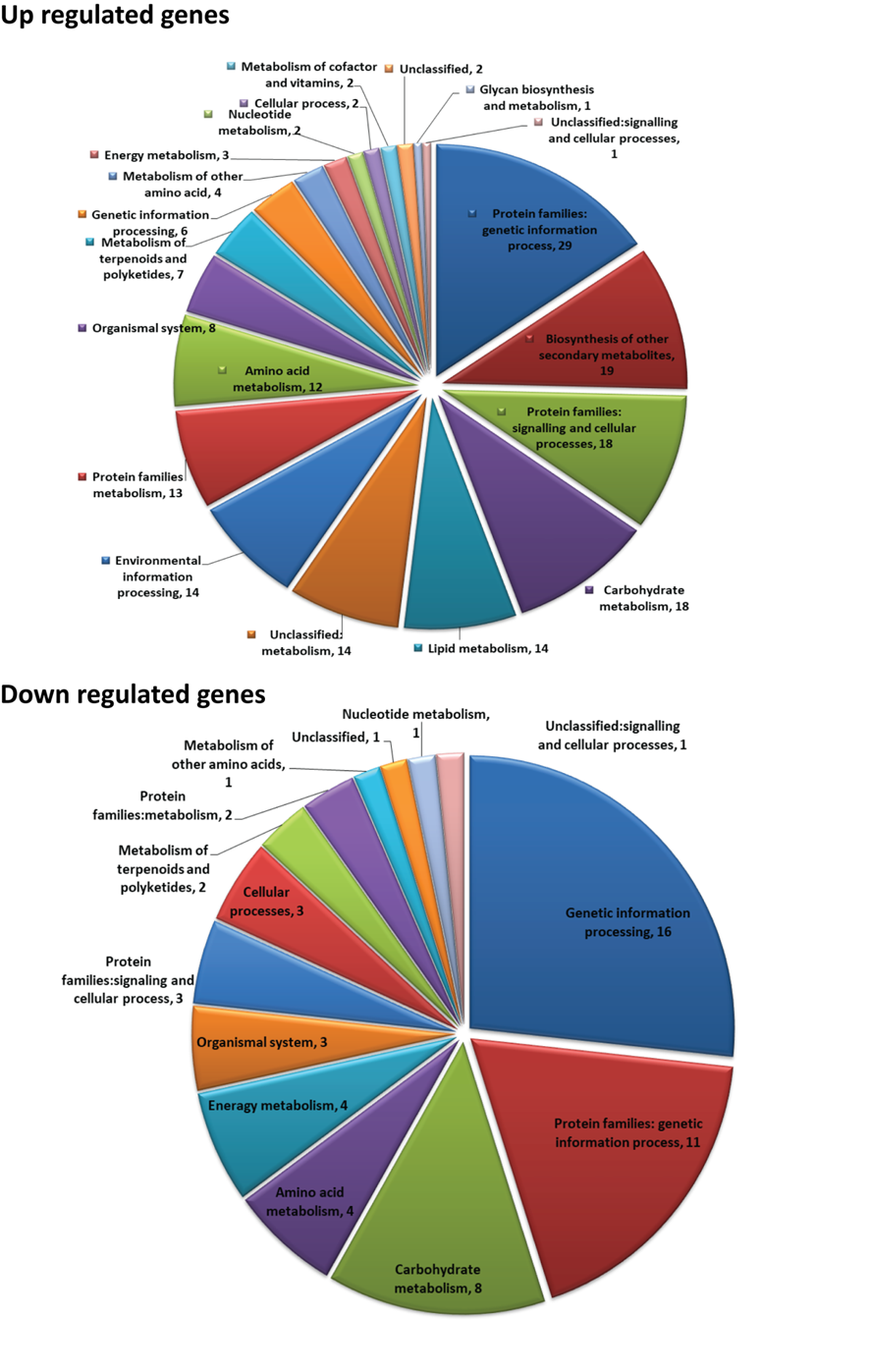


**(B)**

**(A)**

**Figure S4.**

**
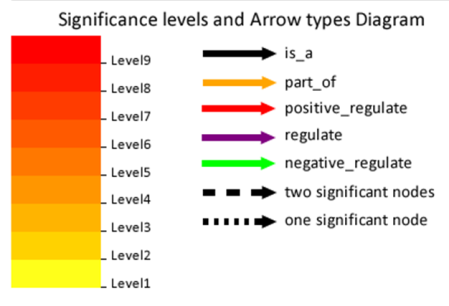
**

**Figure S5.**

**Figure S6**

**(A) OsGW2_Up IR64_Down**


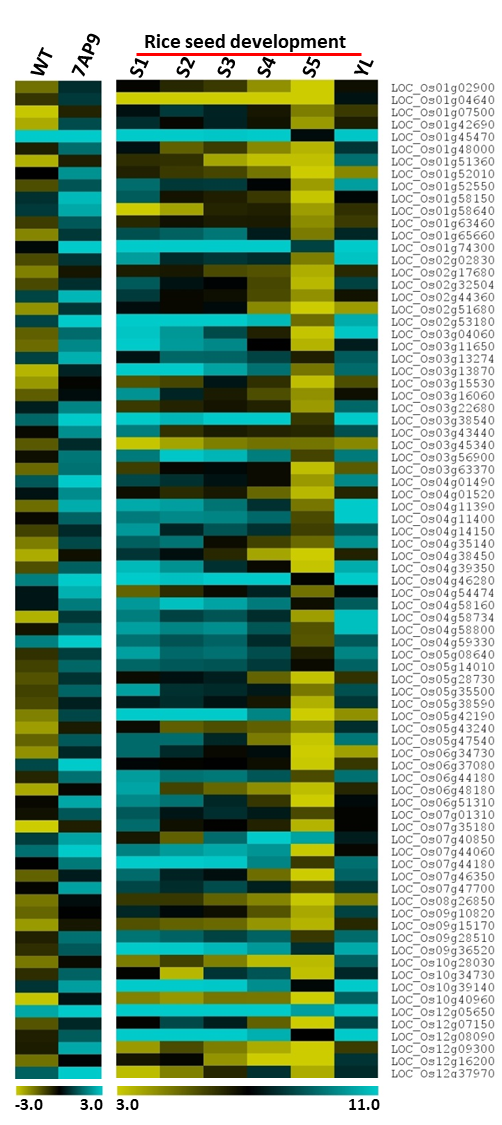


**(B) IR64_Up OsGW2_Down**


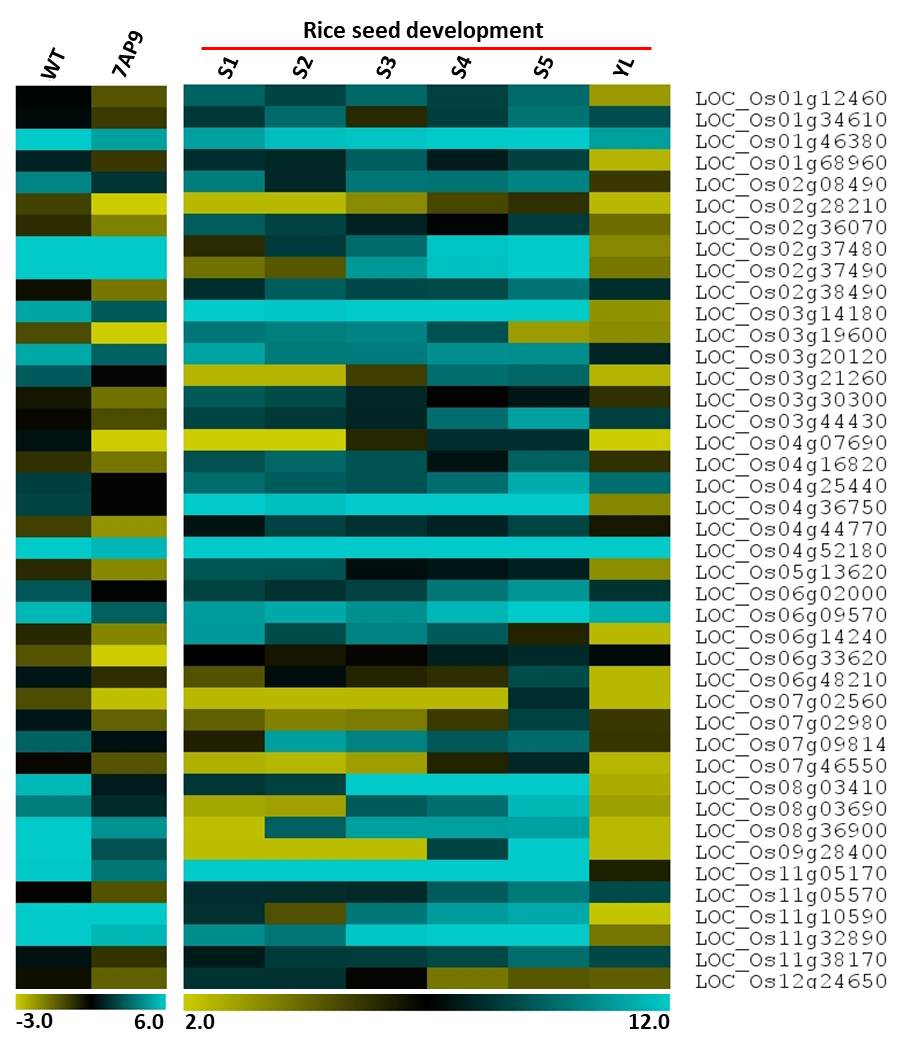


**(C) OsGW2_Down IR64_Down**


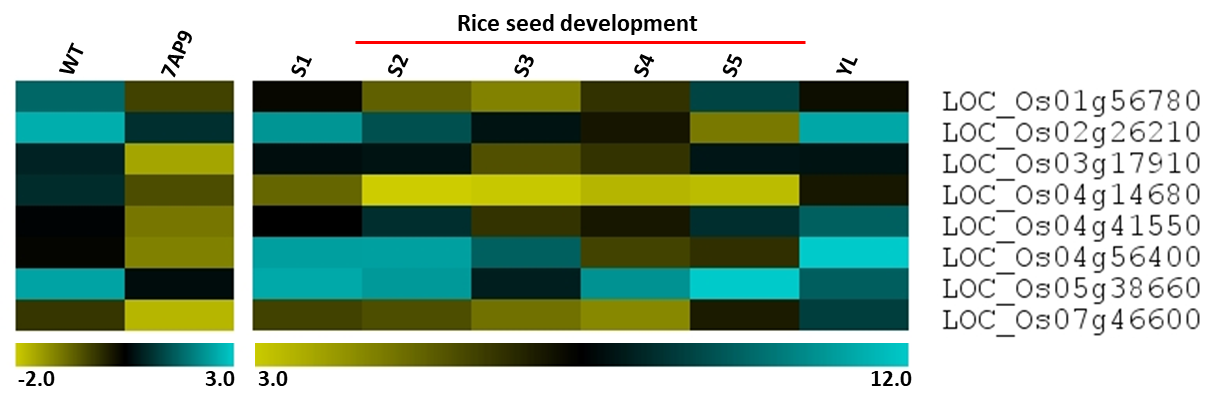

Supplement: Supplementary Figure 1 — Comparison of grain parameters in five different rice genotypes. (A) Grain width, (B) grain length, and (C) grain weight. Error bar represents mean ± SD; n = 100. Grain width and length were measured with WINSEEDLETM. Nipponbare, LGR, Sonasal, IR64, and PB1 represent different rice genotypes. [file Table_1.DOCX]
